# *Cistus albidus* L.—Review of a Traditional Mediterranean Medicinal Plant with Pharmacological Potential

**DOI:** 10.3390/plants12162988

**Published:** 2023-08-18

**Authors:** Daniel Raus de Baviera, Antonio Ruiz-Canales, Enrique Barrajón-Catalán

**Affiliations:** 1 Department of Engineering, Area of Agroforestry, Miguel Hernández University, 03312 Orihuela, Spain; daniel.raus@goumh.umh.es (D.R.d.B.); acanales@umh.es (A.R.-C.); 2 Institute for Research, Development and Innovation in Health Biotechnology, Miguel Hernández University, 03202 Elche, Spain; 3 Department of Pharmacy, Elche University Hospital-FISABIO, 03203 Elche, Spain

**Keywords:** *Cistus albidus*, phytochemistry, pharmacology, polyphenols, terpenes, traditional uses

## Abstract

*Cistus albidus* L. (Cistaceae) is a medicinal plant that has been used therapeutically since ancient times in the Mediterranean basin for its important pharmacological properties. The ability of *C. albidus* to produce large quantities of a wide range of natural metabolites makes it an attractive source of raw material. The main constituents with bioactive functions that exert pharmacological effects are terpenes and polyphenols, with more than 200 identified compounds. The purpose of this review is to offer a detailed account of the botanical, ethnological, phytochemical, and pharmacological characteristics of *C. albidus* with the aim of encouraging additional pharmaceutical investigations into the potential therapeutic benefits of this medicinal plant. This review was carried out using organized searches of the available literature up to July 2023. A detailed analysis of *C. albidus* confirms its traditional use as a medicinal plant. The outcome of several studies suggests a deeper involvement of certain polyphenols and terpenes in multiple mechanisms such as inflammation and pain, with a potential application focus on neurodegenerative diseases and disorders. Other diseases such as prostate cancer and leukemia have already been researched with promising results for this plant, for which no intoxication has been reported in humans.

## 1. Introduction

**Figure 1:**
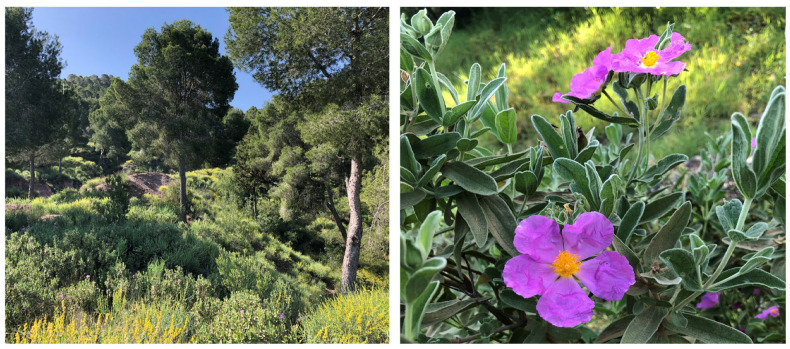
Typical Mediterranean landscape with a predominance of *C. albidus*, called *jaral* (**left**). Detail of flowers and leaves of *C. albidus* in spring (**right**). Images were captured by the authors.

*Cistus albidus* is one of the approximately 20 species of the *Cistus* genus [1]. The genus’ name is derived from the ancient Greek term *kistos* [2]. It is supposed that the name alludes to the woody capsule fruits. Evergreen in its Mediterranean homeland and between 50 and 250 centimeters tall [3], this shrub is called *albidus,* not because of the colour of its flowers, but because its leaves are finely covered with white hair (trichomes) [4]. For its optimal development, it needs calcareous, sandy, or siliceous soils, as found in the *garrigue* of the Mediterranean [5,6]. Here, it can grow in large groups (known as *jarales* in Spanish) (Figure 1) and sometimes invade adjoining areas. It does not make high demands on the soil in terms of nutrients [6], but it needs permeability as it does not tolerate stagnant soils. 

**Figure 2:**
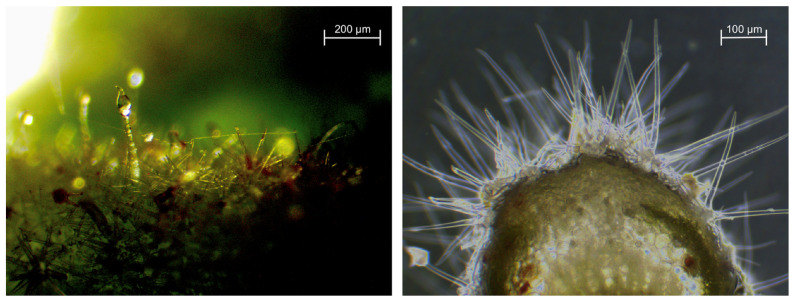
Trichomes: Glandular trichomes with secretion of bioactive compounds on their tops (**left**). Stellar trichomes covering the central vein of a leave (**right**). Images were captured by the authors using a Swift microscope, model SW380T-SC500-5PBC (M TEC USA Inc., Tschertz, TX, USA).

The characteristic feature of *C. albidus* is that its leaves are covered on both sides with dense hairiness made up of a combination of glandular and non-glandular trichomes (Figure 2). The glandular trichomes can be stellate or solitary; they are elongated and produce and secrete a resin rich in metabolites of pharmacological interest [7], such as flavonoid aglycones, glycosides, and terpenoids, including the characteristic labdane-type diterpenes described in Table 1 and Table 2. It is because of these compounds that this species was traditionally used in popular medicine according to its pharmacological action as an anti-inflammatory, antimicrobial, antinociceptive, and sedative [8,9,10]. 

Currently, its use as a medicinal plant is vestigial; however, its phytochemical composition, especially the combination of some terpenoids and polyphenols, makes it a promising species with many pharmacological activities. Pharmacological studies on extracts of *C. albidus* have shown their antioxidant, antibacterial, antifungal [5,11,12], antinociceptive [13], and anticarcinogenic [5,14] activity, in addition to other potential activities discussed later in this review. 

Nowadays, however, *C. albidus* is known more for its ability to withstand severe summer stress situations to repopulate degenerate areas [15,16,17,18] than for its important pharmacological properties. Due to its ability to produce large amounts of secondary metabolites, it becomes an attractive model for elucidation of the biosynthetic pathways involved in resisting climatic adversities [19,20,21,22]. The pathways that lead to the production of terpenes and polyphenols have been investigated on many occasions, and the substances have been analyzed in isolation. However, in the author´s opinion, the potential of *C. albidus* does not lie in the independent substances that compose it, but in the interaction among them. 

## 2. Distribution

Cistus is a characteristic genus of the Mediterranean flora. The rockrose family has spread throughout the western Mediterranean. The genus is now believed to comprise approximately 20 species, of which 16 are native to Europe [1]. Some species of Cistus are endemic, while others are widespread in the Iberian Peninsula, the Canary Islands, Northwest Africa, Italy, Greece, France, or Turkey [1,21]. 

**Figure 3:**
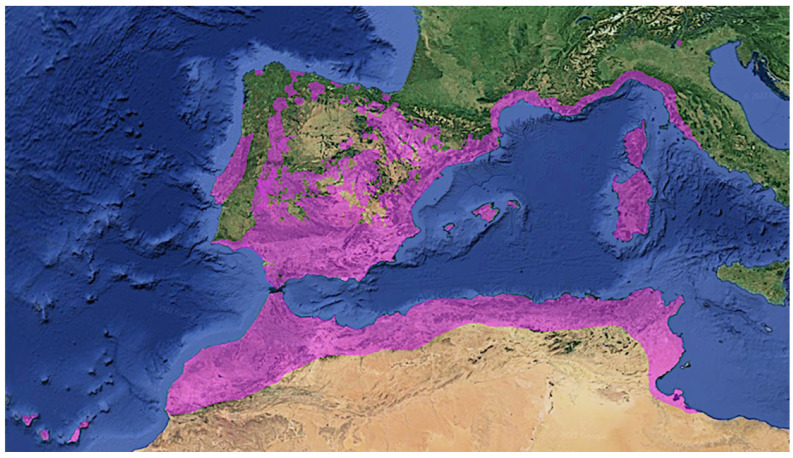
Natural distribution of *Cistus albidus* in the western Mediterranean basin (distribution map compiled using Google Earth (https://www.google.com/earth/download/, accessed on 19 April 2022). Highlighted in pink, the known natural distribution of *C. albidus*.

The often-coexisting species *C. albidus* L., *C. crispus* L., *C. creticus* L., and *C. heterophyllus* Desf. are native only to the western part of the Mediterranean, with the exception of *C. criticus*, which also reaches the eastern Mediterranean and the Black Sea coasts and the Crimean peninsula [18]. As can be seen in Figure 3, the currently known distribution of *C. albidus* includes the Iberian Peninsula, the Canary Islands, and the western Mediterranean. The northernmost population, by far, of *C. albidus* is found near Lake Garda in Italy [23]. It grows from sea level to 1300 m [24] and tolerates temperatures down to −12 °C, having an even lower thermal threshold [23].

## 3. Systematics

**Figure 4:**
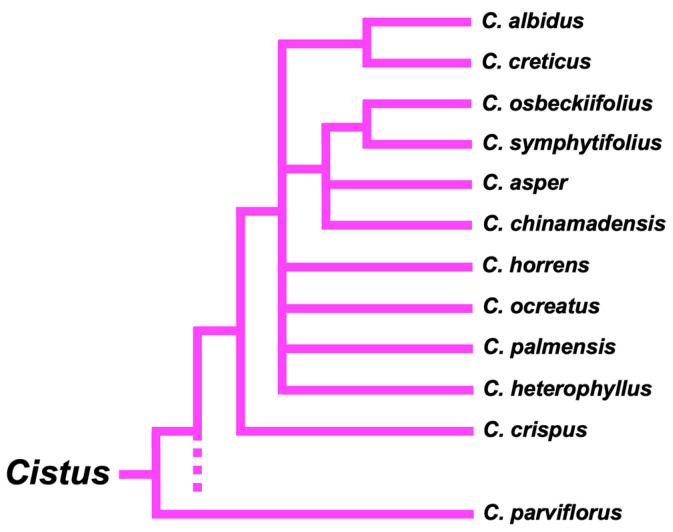
Systematics of the pink-purple flowered species of *Cistus* genus based on Guzmán and Vargas [1] and Civeyrel et al. [23].

Cistus is one of eight genera found within the rockrose family (Cistaceae), which is a part of the Malvales order. The Cistaceae encompasses approximately 180 species [25]. Within this family, genera that are native to the Mediterranean basin include Cistus, Halimium, Fumana, Tuberaria, and Helianthemum, while Crocanthemum, Hudsonia, and Lechea are endemic to the American continent [26]. The Cistus genus is further categorized into the subgenus Cistus, which includes nine species. In the Iberian Peninsula, this subgenus contains species such as C. albidus L., C. crispus L., C. creticus L., and C. heterophyllus Desf., which often coexist. Among the species C. crispus, C. creticus, and C. heterophyllus, C. albidus represents one of the four paraphyletic species within the subgenus Cistus [18] (Figure 4). DNA sequence and pollen analysis showed a close evolutionary relationship of C. creticus and C. albidus, with C. crispus and C. heterophyllus more distantly related taxa [1,11,21]. Calculations have determined that the species C. albidus, C. creticus, and C. heterophyllus separated 0.19 Ma ago [21]. 

All representatives of this genus have a similar chromosome number (2n = 18), which has facilitated hybridization between species [11]. Nowadays, more than twenty natural interspecific hybrids are known, in addition to numerous cultivars of hybrid origin used for ornamental purpose [1,18]. As a consequence of this scenario, although the main characteristics of C. albidus are easily identifiable, they can be confused with these species due to their similarity of characteristics, especially the inflorescences.

## 4. Botanical Characteristics

From the morphological point of view, *C. albidus* is characterized by having five sepals, five pink to purple petals, up to 150 yellow stamens, pollen with a thin exine up to 1.4 μm thick, a long style that reaches or exceeds the stamens, and three cellular polysperm capsules. The hermaphroditic, actinomorphic, and hypogynous flowers, which appear from February to July, normally reach a diameter of four to six centimeters and develop individually or in umbels of usually five to seven [25]. The five sepals are ovate-lanceolate and hairy. The five petals, on the other hand, are delicate and slightly wrinkled. The flowers open in the morning and after a few hours the plant loses its petals. The flowers rarely last more than a day. A single adult bush can produce more than 1000 flowers per flowering period, depending on age [27].

The ovate-lanceolate leaves are arranged opposite each other and are usually 20–50 mm long and 8–30 mm wide; however, specimens with leaves exceeding 100 mm long and more than 50 mm wide have also been found by the authors. They are sessile, with a smooth margin, but may occasionally have a slightly wavy edge. Foliar veins are composed of three to five principal veins with a strong central vein. Adaxis veins are sunken, while veins on the abaxial surface are raised (Figure 2).

Due to its morphological adaptation to an extremely dry climate within Mediterranean regions and a pronounced resistance to abiotic stress in general, *C. albidus* could be considered a malacophilous xerophyte. During dry periods, these plants reduce the growth of relatively long and wide hairy leaves until only short, narrow leaves remain. At the same time, the hairiness (trichomes) of the leaves of these plants is increased. In general, these processes significantly reduce respiration, which is an effective protection mechanism against dehydration [28,29].

### Vegetative Development

*C. albidus* has developed mechanisms that allow it to withstand severe summer droughts. These mechanisms consist in part of a reduction in leaf area and angle [28], combined with an increase in root mass per leaf area and modification of stomatal conductance [30]. On the other hand, the link between tocopherols and jasmonates appears to be primarily responsible for the regulation of biotic and abiotic stress responses [20]. It was observed that, under water stress, *C. albidus* increases enzymes related to redox homeostasis, such as oxidized ascorbate reductase, glyoxalase, superoxide dismutase, and isoflavone reductase, which was related to a reduction in oxidative stress in *C. albidus* exposed to drought and the ability to recover quickly after re-irrigation [19]. The interaction of these mechanisms allows this species to resist the adverse Mediterranean climate conditions with only little photoinhibition [20]. 

Furthermore, *C. albidus* belongs to the nanophanerophytes (a subgroup of phanerophytes). These are plants whose overwintering buds are above the level of the snow cover. In contrast to the macrophanerophytes, they do not rise above the level of the surrounding vegetation. They are therefore partially sheltered from the wind. Due to this fact, a more than 2 m high specimen was discovered in the Valle y Carrascoy Regional Park in Murcia, surrounded by shrubs up to 3 m high.

The vegetative development of this species, which normally lives for about 14 years [31] and was found to reach 17 [32] or even 25 years [27], is characterized by two types of lateral shoots, dolichoblasts and brachyblasts. Dolichoblasts are long shoots with large leaves, which are produced when climatic conditions are benign (availability of water and absence of frost), which is usually between the end of February to May and from September to December. Brachyblasts are short shoots that develop throughout the year in the axils of leaves of dolichoblast shoots [33]. The fall of the leaves of this marcescent species is acropetic. 

Sexual reproduction begins at about the age of one year. The flowers of this partially self-incompatible species [34] normally last around 12 h but may last up to two days on the plant, especially in rainy weather with high relative air humidity. This seems to be due to the fact that the apoidea, its main pollinators [34], do not fly in humid environmental conditions. It has been further shown that zeatin is the substance that modulates the speed of floral development, depending on the age of the plants [22].

Fruiting takes place from May to August. The capsules contain an average of about 80 seeds, with exceptions found by the authors from fewer than 10 seeds to more than 140, and generally mature from August to December [33,34], but in warmer regions such as the Spanish Levant, for example, the first capsules usually mature at the end of May. Mature capsules spread their seeds close to parent plants as they lack expansion mechanisms. Studies suggest that *C. albidus* seeds experience a combination of physical and physiological dormancy [35]. Although physical dormancy was broken and water was available, the seeds seemed able to partially control their dormancy and germination capacity [35]. Under optimal conditions, germination takes between five and ten days and is epigeal [36]. 

Like other plants typical of fire-prone regions, *C. albidus* is generally considered pyrophytic [37], especially since the heat generated by fire is thought to facilitate breaking physical dormancy due to the hard seed coat [31,38,39]; thus, it is one of the first shrubs to emerge after a fire. Development is therefore rapid within the first five years and then progressively slows down [31]. However, the scarification of the seeds, by soil particles, (through dragging by water flow) also softens the hard cover of the seeds, making them permeable to water [40], thus overcoming physical dormancy [40,41]. In addition, it is common to find this species along the edges of watercourses with temporary flows. This seems to be its dispersal strategy since forest fires do not facilitate the spatial expansion of this species. Very specific conditions must be met for the seeds to viably survive forest fires and also break the integument. For this reason, *C. albidus* could be considered an opportunistic pioneer plant. 

Regarding the influence of the soil on the development of *C. albidus*, together with *C. creticus*, it is the only taxon of the purple-pink clade capable of growing independently in calcareous and acidic soils. However, *C. albidus* grows best on calcareous soils in Mediterranean climates [5,42]. Studies reported that no significant qualitative or quantitative differences were found in the polyphenolic profile between the cultivation of *C. albidus* in different types of soil [5,6], while the concentration of terpenes was influenced by soil conditions, showing lower yields in calcareous soils [43]. However, higher concentrations of polyphenols were not associated with lower soil fertility [44]. This suggests that the genetic influence of this species on the biosynthesis of phytochemicals may be stronger than the influence of soil parameters. This was confirmed by a recent study, where *C. albidus* exhibited a low translocation of Pb and Cd to aerial parts from heavy-metal-contaminated soil [17], making this species also suitable for plantations under problematic soil conditions.

## 5. Phytochemical Constituents

The main constituents of *C. albidus*-derived products belong to the groups of terpenes and polyphenols. Other organic compounds have also been detected. To date, more than 200 secondary metabolites have been reported in *C. albidus* samples. In this review, 153 terpenoids, including 31 monoterpenes, 109 sesquiterpenes, 9 diterpenes, and 3 tetraterpenes, and their respective derivatives were found. In addition, 58 polyphenols, including 19 phenolic acids, 17 flavonols, 11 flavanols, 3 ellagitannins, 3 anthocyanins, 2 flavones, 1 anthocyanidin, 1 flavanone, and 1 hydrolysable tannin, were found. Moreover, 8 fatty acids, 7 alkanes, and various other compounds were found in the literature and discussed in the present study. 

The irregular presence of reported compounds–analyses were often very heterogeneous—is possibly due to seasonal variation and the analytical methods used. Secondary metabolites reported so far have been determined under a wide variety of conditions, making it sometimes difficult to compare among the studies. For example, some metabolites, such as punicalagin derivatives or some diterpenes, were only found in a small number of studies [5,11]. The results depend on multiple factors, such as the method of analysis, the season and hours of the day of collection, and the type of processing, among others, and some of these factors are not detailed in many studies. However, on the other hand, there are a number of compounds that practically all authors found in significant amounts, being thus characteristic of this species. These more common compounds will be highlighted in the following sections. 

The various compound structures detected in *C. albidus* are described below, headed by the terpenes and paying particular attention to the essential oils (mono- and sesquiterpenes). Phenylpropanoids detected in essential oils are also briefly discussed. Furthermore, di- and tetraterpenes are described. The second large group of substances, polyphenols, are examined more closely, especially flavonols, flavanols, ellagitannins, and phenolic acids. The carbonyl compounds and alkanes found so far are then listed. Finally, proven phytohormones and various fatty acids are described. However, the latter is not part of the secondary metabolism but is included here for its supposed importance as a potential bioavailability enhancer.

### 5.1. Terpenes

#### 5.1.1. Mono- and Sesquiterpenes from the Essential Oils 

Terpenes, a very large but heterogeneous group of naturally occurring secondary metabolites in *C. albidus*, represent the group with the most compounds identified in this species. Among them, the most abundant were the sesquiterpenes; they were, in addition, the largest class of identified compounds in *C. albidus*. While monoterpenes were found to contribute, to a certain extent, together with aldehydes, to the characteristic odor of this species (they are among the main components of floral aromas), the sesquiterpenes play a signaling role in the defense mechanisms of this species, acting as herbivore repellents or through the attraction of predators. Diterpenes are also synthesized for defense purposes and serve as precursors for vitamins and hormones such as tocopherols and gibberellins. Finally, tetraterpenes contribute to the pigmentation of flowers and fruits, playing an essential role in the pollination and distribution of seeds. As an antioxidant, it protects *C. albidus* from oxidative stress caused by adverse growing conditions [30]. 

The ISO definition for essential oils is “Product obtained from a natural raw material of plant origin, by steam distillation, by mechanical processes from the epicarp of citrus fruits, or by dry distillation, after separation of the aqueous phase—if any—by physical processes” [45].

Essential oils from fresh aerial parts of *C. albidus* were generally obtained in low yields of 0.01–0.1% (*w*/*w*) [46,47,48] by steam distillation. The seeds, in particular, contain very small amounts of essential oil, sometimes insufficient to be analyzed, with a yield of less than 0.01% [48]. The concentration of terpenes depends fundamentally on soil conditions (the more calcareous, the lower the yield), climatic factors, and the season [43]. Analysis of seasonal variation in terpene composition shows strong interannual variability, with the highest emission rates in autumn and spring and the lowest in summer and winter, leading to maximum values of stored terpenes in autumn and winter, while the spring and summer values showed minimum levels [47]. Table 1 shows the list of the terpenoids identified in *C. albidus* samples, including information about their previously published pharmacological activity. The compounds most frequently found in the analyses of the extracted terpenes (*w*/*w*), and therefore representative of this species, whether in leaves, pollen, flowers, flowering tops, or stems, are *α*-zingiberene (7.4–20.7%), aromadendrene (1.0–10.6%), ar-curcumene (8.3–13.2%), and germacrene D (1.0–7.9%) [46,48,49,50,51,52,53]. Among the terpenes in leaves, flowering tops, and flowers, monoterpenes were only present in small quantities and sometimes only in traces, while sesquiterpenes were the most abundant [46,48,49].

**Table 1:** Identified terpenes in *Cistus albidus.* Table includes the structure for each compound along with the references in which it was identified and its pharmacological activity (including the references for this activity). 🌱: aerial parts, including leaves and twigs; ✿: flowering tops, flowers, petals, and sepals; 𐩕: pollen. n/a: reliable data are not available.

No	Compound	Structure/Class	Presence in	Analytical Reference	Pharmacology
1	*cis-α*-Bergamotene	polycyclic monoterpene hydrocarbon	🌱✿	[48,49,53]	n/a
2	*trans-α*-Bergamotene		🌱	[47,48]	
3	Borneol	polycyclic monoterpene alcohol	🌱	[52]	blood brain barrier (BBB) permeability improvement, intercellular tight junction (TJ) loosening [54]
4	Camphor	oxygenated polycyclic monoterpene	🌱	[50]	analgesic, antinociceptive [55]; antimicrobial, antiviral [56]; anticancer [57]; antitussive [58]; skin penetration enhancer [59];
5	Carene	polycyclic monoterpene hydrocarbon	🌱✿	[52]	antiviral [60]; enhances bone mineralization [61]; anti-inflammatory [62];
6	Carvacrol	monocyclic monoterpene alcohol	🌱✿	[49]	antibacterial (64); antifungal [63]; antioxidant [64]; anticancer [65]; anti-inflammatory, analgesic [66]; antiobesity [67]; hepatoprotective [68]; spasmolytic [69]; vasorelaxant [70];
7	*β*-Cyclocitral	oxygenated monocyclic monoterpene	🌱	[53]	n/a
8	*p*-Cymene	monocyclic monoterpene hydrocarbon	🌱	[49,50]	anti-inflamatory, antinociceptive, antioxidant [71]; antidiabetic [72];
9	*p*-Cymenene	monocyclic monoterpene hydrocarbon	🌱✿	[50]	n/a
10	Isobornyl formate	oxygenated polycyclic monoterpene	🌱	[50]	n/a
11	(D-)Limonene	monocyclic monoterpene hydrocarbon	🌱✿	[47,48,49,50]	anticancer, anticholesterol [73]; antidepressant [74];
12	Linalool	acyclic monoterpene alcohol	🌱✿	[51,52]	antibacterial, antifungal [75]; anxiolytic [76]; anticancer, antioxidant [77]; analgesic [78]; anti-inflammatory [79];
13	*cis*-linalool oxide	oxygenated heteromonocyclic monoterpene		[51]	n/a
14	Myrcene	acyclic monoterpene hydrocarbon	🌱	[49,51]	analgesic, antinociceptive [80];
15	Neryl acetate	acyclic monoterpene hydrocarbon	🌱✿	[53]	n/a
16	(E)-Ocimene	acyclic monoterpene hydrocarbon	🌱✿	[49]	anticancer [81]; anticonvulsant [82];
17	(*Z*)-*β*-Ocimene			[49]	anticancer [81]; antibacterial [83];
18	*α*-Phellandrene	monocyclic monoterpene hydrocarbon	🌱	[49]	antifungal [84]; antidepressant [85]; anti-inflammatory, antihyperalgesic [85,86], analgesic, antinociceptive [86]; anticancer [87];
19	*β*-Phellandrene		✿	[49]	n/a
20	*α*-Pinene	polycyclic monoterpene hydrocarbon	🌱	[47,49,51]	antifungal, anti-inflammatory, antioxidant [88]; anticancer [89]; anti-Leishmania [90]; gastroprotective [91,92]; antibacterial [93]; antiviral [94]; neuroprotective [95];
21	*β*-Pinene		🌱✿	[47]	anticancer [96]; antimicrobial [97]; gastroprotective [92]; neuroprotective [98];
22	Piperitone	oxygenated monocyclic monoterpene	𐩕	[49]	n/a
23	Sabinene	polycyclic monoterpene hydrocarbon	🌱	[49]	n/a
24	*cis*-Sabinene hydrate	oxygenated polycyclic monoterpene		[49]	
25	Safranal	oxygenated monocyclic monoterpene	🌱	[53]	antioxidant [99]; antimicrobial [100]; anticonvulsant [101]; antidepressant, anxiolytic [102]; gastroprotective [103];
26	*α*-Terpinene	monocyclic monoterpene hydrocarbon	🌱	[49]	antioxidant [104]; antimicrobial [105];
27	Δ-Terpinene		🌱✿	[52]	n/a
28	*γ*-Terpinene		✿	[49]	antimicrobial [105];
29	*α*-Terpineol	monocyclic monoterpene alcohol	✿ 𐩕	[49,50,52,53,106]	antioxidant, anticancer, antinociceptive, anticonvulsant, sedative, antibronchitis, antihypertensive, vasorelaxant, cardioprotective [107];
30	4-Terpineol		🌱	[49,53]	antimicrobial [108]; gastroprotective [109];
31	Thymol	monocyclic monoterpene hydrocarbon	🌱✿	[49,50]	anti-inflammatory, antioxidant, antimicrobial, immunostimulatory, anticancer [110]; cardioprotective [111]; antihypertensive [70]; antihyperglycemic [112]; antinociceptive [113]; gastroprotective [114]; anxiolytic [115];
32	Abscisic acid	oxygenated monocyclic sesquiterpene	🌱	[116,117]	antidiabetic [118]; antinociceptive [119];
33	*α*-Amorphene	polycyclic sesquiterpene hydrocarbon	🌱	[50,53]	n/a
34	Aromadendrene	polycyclic sesquiterpene hydrocarbon	🌱✿	[47,48,53]	antimicrobial [120];
35	allo-Aromadendrene	polycyclic sesquiterpene hydrocarbon	🌱✿	[6,43,46,48,49,50,51,52,53]	
36	allo-Aromadendrene epoxide	oxygenated polycyclic sesquiterpene	🌱	[53]	
37	Bisabola-2,10-diene(1-9)oxide	oxygenated polycyclic sesquiterpene	🌱	[53]	n/a
38	*β*-Bisabolene	monocyclic sesquiterpene hydrocarbon		[48]	anticancer [121]
39	epi-*α*-Bisabolol	monocyclic sesquiterpene alcohol	🌱✿	[46,48,49,53]	anti-inflammatory [122]; antimicrobial [123]; anticancer [124];
40	*α*-Bisabolol		🌱	[43,53]	n/a
41	*β*-Bisabolol		🌱✿	[48]	n/a
42	*β*-Bourbonene	polycyclic sesquiterpene hydrocarbon	🌱✿	[6,43,46,47,49,50,51,52,53]	n/a
43	1,5-di-epi-Bourbonene (*α* or *β*)		🌱	[53]	
44	Bulnesol	polycyclic sesquiterpene alcohol	🌱✿	[49]	n/a
45	Cadalene	polycyclic sesquiterpene hydrocarbon	🌱	[53]	n/a
46	Cadina-1,4-diene	polycyclic sesquiterpene hydrocarbon	🌱✿	[48,53]	n/a
47	*α*-Cadinene	polycyclic sesquiterpene hydrocarbon	🌱	[50,53]	n/a
48	*cis-γ*-Cadinene		🌱✿𐩕	[49]	
49	*trans-γ*-Cadinene		🌱	[46]	
50	*γ*-Cadinene		🌱	[51,52,53]	
51	*δ*-Cadinene		🌱✿𐩕	[43,46,48,49,50,52,53]	
52	*α*-Cadinol	polycycylic sesquiterpene alcohol	🌱✿	[43,48,49,53]	antifungal [125];
53	T-Cadinol			[46,49,53,126]	anticancer [127]
54	*α*-Calacorene	polycyclic sesquiterpene hydrocarbon	🌱	[50,53]	n/a
55	*β*-Calacorene			[53]	
56	Calamenene	polycyclic sesquiterpene hydrocarbon	🌱	[50,53]	anticancer [127];
57	Caryophylladienol I	polycyclic sesquiterpene alcohol	🌱	[53]	n/a
58	Caryophylladienol II			[53]	
59	*β*-Caryophyllene	polycyclic sesquiterpene hydrocarbon	🌱✿𐩕	[6,43,46,47,48,49,50,51,52,53]	antioxidant, antimicrobial, antitumor, anticancer [128]; anti-inflammatory, neuroprotective [129]; anxiolytic, antidepressant [130]; anticonvulsant [131]; analgesic [132];
60	*β*-Caryophyllene epoxide	oxygenated polycyclic sesquiterpene	🌱	[43,46,48,49,53]	anticancer, analgesic [132];
61	Caryophyllenol II	polycyclic sesquiterpene alcohol	🌱	[53]	n/a
62	8,14-Cedranoxide	oxygenated sesquiterpene	🌱	[46]	n/a
63	*α*-Cedrene	polycyclic sesquiterpene hydrocarbon	🌱	[47]	n/a
64	*α*-Copaene	polycyclic sesquiterpene hydrocarbon	🌱	[6,46,47,49,50,52,53]	antioxidant, anticancer [133]; neuroprotective [134];
65	*β*-Copaene		🌱✿𐩕	[46,50,53]	
66	*α*-Corocalene	polycyclic sesquiterpene hydrocarbon	🌱	[53]	n/a
67	*α*-Cubebene	polycyclic sesquiterpene hydrocarbon	🌱✿	[6,49,53]	antioxidant, neuroprotective [135]; antimicrobial [136]; anti-inflammatory [137];
68	*β*-Cubebene		🌱	[6,46,49]	
69	Cubebol	polycyclic sesquiterpene alcohol	🌱	[53]	n/a
70	4-epi-Cubebol			[53]	
71	1,10-di-epiCubenol	polycyclic sesquiterpene alchohol	🌱✿	[46,49,53]	n/a
72	Cubenol		🌱	[53]	
73	1-epi-Cubenol		🌱✿	[46,49,53]	
74	ar-Curcumen-15-al	oxygenated monocyclic sesquiterpene	🌱	[46,53]	n/a
75	ar-Curcumene	monocyclic sesquiterpene hydrocarbon	🌱✿	[6,43,46,47,48,49,50,51,52,53,126]	n/a
76	*β*-Curcumene		✿	[48]	
77	*γ*-Curcumene		🌱✿	[48,49]	
78	Curcuphenol	monocyclic sesquiterpene alcohol	🌱✿	[48,53]	anticancer [138];
79	Cyclosativene	polycyclic sesquiterpene hydrocarbon	🌱✿	[49]	n/a
80	Dehydrosesquicineole	oxygenated polycyclic sesquiterpene	🌱	[53]	n/a
81	Bicyclo-Elemene	polycyclic sesquiterpene hydrocarbon	🌱	[50]	n/a
82	*β*-Elemene	monocyclic sesquiterpene hydrocarbon	🌱	[48,53]	anticancer, anti-inflammatory [139];
83	*γ*-Elemene		🌱	[48]	
84	*δ*-Elemene		🌱✿	[48,49,50]	
85	Elemol	monocyclic sesquiterpene alcohol	🌱	[43,48,49]	n/a
86	*β*-Eudesma 4(15), 7 dien-1*β*-ol	polycyclic sesquiterpene alcohol	🌱	[53]	n/a
87	*α*-Eudesmol		🌱	[46]	neuroprotective [140];
88	*β*-Eudesmol		🌱✿	[46,48]	anti-allergic, anti-inflammatory [141]; anticancer [142];
89	*γ*-Eudesmol		🌱✿	[48,49]	n/a
90	10-epi-*γ*-Eudesmol			[48,49]	n/a
91	Kunseaol	monocyclic sesquiterpene alcohol	🌱	[53]	n/a
92	Bicyclo-Germacrene	polycyclic sesquiterpene hydrocarbon	🌱	[53]	n/a
93	Germacrene B	monocyclic sesquiterpene hydrocarbon	🌱	[47,48]	
94	Germacrene D		🌱✿𐩕	[6,43,47,48,49,51,52,53]	anticancer [143]; anti-inflammatory, analgesic [144]; antioxidant [145];
95	Iso-Germacrene D		🌱	[53]	
96	*β*-Germacrenol	monocyclic sesquiterpene alcohol	🌱	[53]	n/a
97	Globulol	polycyclic sesquiterpene alcohol	🌱✿	[46,48]	n/a
98	*α*-Guaia-6,10(14)-diene-4*β*-ol	polycyclic sesquiterpene alcohol	🌱	[53]	n/a
99	Guaiene	polycyclic sesquiterpene hydrocarbon	🌱	[46]	n/a
100	Guaiol	polycyclic sesquiterpene alcohol	🌱✿	[48,49]	n/a
101	*α*-Gurjunene	polycyclic sesquiterpene hydrocarbon	🌱✿	[47,49,51,52]	n/a
102	*β*-Gurjunene			[49]	
103	*β*-Himachalene	polycyclic sesquiterpene hydrocarbon	✿ 𐩕	[49]	n/a
104	*α*-Humulene	monocyclic sesquiterpene hydrocarbon	🌱✿	[6,43,46,47,48,49,51,52,53]	antitumor, anti- inflammatory, antimicrobial [146];
105	Iso-Calamendiol	polycyclic sesquiterpene alcohol	🌱	[53]	n/a
106	Iso-Italicene	polycyclic sesquiterpene hydrocarbon	🌱	[46]	n/a
107	Juniper camphor	polycyclic sesquiterpene alcohol	🌱✿	[49]	n/a
108	Ledol	polycyclic sesquiterpene alcohol	🌱	[53,106]	n/a
109	*α*-Longipinene	polycyclic sesquiterpene hydrocarbon	🌱	[46]	n/a
110	*cis*-Muurola-4(14),5-diene	polycyclic sesquiterpene hydrocarbon	🌱✿	[49,53]	n/a
111	*α*-Muurolene			[6,48,49,50,52]	
112	*γ*-Muurolene			[46,49,53]	
113	14-hydroxi-α-Muurolene	polycyclic sesquiterpene alcohol	🌱	[46]	n/a
114	*α*-Muurolol		🌱✿	[49]	
115	epi-*α*-Muurolol			[48]	
116	T-Muurolol			[43,46,49,49,53]	
117	E-Nerolidol	acyclic sesquiterpene alcohol	🌱✿	[48]	antihyperlipidemic, anti-inflammatory, anti-uterine fibroids [147]; anticancer [148];
118	(E)-Nuciferol	moncyclic sesquiterpene alcohol	🌱	[53]	n/a
119	*β*-Oplopenone	oxygenated polycyclic sesquiterpene	🌱	[53]	n/a
120	Salvial-4(14)-en-1-one	oxygenated polycyclic sesquiterpene	🌱	[53]	n/a
121	*α*-Santalene	polycyclic sesquiterpene hydrocarbon	🌱	[50]	n/a
122	cis-*α*-Santalol	polycyclic sesquiterpene alcohol	🌱	[46]	antihyperglycemic, antioxidant [149];
123	Selin-11-en-4-*α*-ol	polycyclic sesquiterpene alcohol	🌱	[106]	anxiolytic, sedative [150];
124	Selina-3,7(11)-diene	polycyclic sesquiterpene hydrocarbon	🌱	[50]	n/a
125	*α*-Selinene	polycyclic sesquiterpene hydrocarbon	🌱	[50]	n/a
126	*β*-Sesquiphellandrene	monocyclic sesquiterpene hydrocarbon	🌱✿	[43,47,49,53]	anticancer [151]; antioxidant [152];
127	*trans*-Sesquisabinene hydrate	polycyclic sesquiterpene alcohol	🌱	[48]	n/a
128	Shyobunone	oxygenated monocyclic sesquiterpene	🌱	[6,53]	neuroprotective, acetyl-cholinesterase inhibition [153];
129	6-epi-Shyobunone			[53]	
130	iso-Shyobunone			[53]	
131	Spathulenol	polycyclic sesquiterpene alcohol	🌱✿	[46,48,49,53]	neuroprotective [154]; antibacterial, antioxidant, anti-inflammatory, anticancer [155];
132	Spathulenol isomer		🌱	[48]	
133	ar-Turmerol	monocyclic sesquiterpene alcohol	🌱✿	[46,48]	n/a
134	Valerianol	polycyclic sesquiterpene alcohol	🌱✿	[49]	n/a
135	Viridiflorol	polycyclic sesquiterpene alcohol	🌱✿	[48,53]	anti-arthritic, analgesic, antinociceptive [156]; anticancaer [157]; antioxidant, antibacterial, anti-inflammatory [158];
136	Xanthorrhizol	monocyclic sesquiterpene alcohol	🌱	[46,53]	anticancer [159]; antimicrobial, antibacterial [160]; antihypolipidemic [161]; anti-inflammatory [162];
137	*α*-Ylangene	polycyclic sesquiterpene hydrocarbon	🌱	[50]	n/a
138	*β*-Ylangene		🌱	[53]	
139	*α*-Zingiberene	monocyclic sesquiterpene hydrocarbon	🌱✿𐩕	[6,43,46,47,48,49,50,51,52,53]	analgesic, neuroprotective [163]; anticancer [164]; anti-inflammatory [165];
140	Zingiberenol	monocyclic sesquiterpene alcohol	🌱	[53]	n/a
141	15,16-Dinorlabd-8(20)-en-13-one	oxygenated diterpene	🌱	[106]	n/a
142	Geranyl-*p*-cymene	monocyclic diterpene hydrocarbon	🌱	[53]	n/a
143	Geranyl *α*-terpinene	monocyclic diterpene hydrocarbon	🌱	[53]	n/a
144	Geranyl linalool	acyclic diterpene alcohol	🌱	[53]	n/a
145	Gibberellin	oxygenated polycyclic diterpene	🌱	[29]	n/a
146	Manool	polycyclic diterpene alcohol	🌱	[106]	antioxidant, anti-inflammatory [166]; anticancer [167]; antihypertensive [168]; cardioprotective [169];
147	Manoyl oxide	polycyclic oxygenated diterpene	🌱	[126]	anticancer [170];
148	13-epi-Manoyl oxide			[47,53,126]	
149	Methyl Neoabietate	oxygenated polycyclic diterpene	🌱	[47]	n/a
150	lutein	oxygenated polycyclic tetraterpene	🌱	[29]	antioxidant, anti-inflammatory, neuroprotective [171]; anticancer [172]; hepatoprotective [173]; cardiopreotective [174];
151	neoxanthin	oxygenated polycyclic tetraterpene	🌱	[29]	antihyperlipidemic [175];
152	zeaxanthin	oxygenated polycyclic tetraterpene	🌱	[29]	n/a

The following quantities refer to the *w*/*w* of the extracted oil. Oxygenated sesquiterpenes ranged from 44.8% in aerial parts (twigs, leaves, flowers) [46] to 67.1% in leaves [49]. Hydrocarbon sesquiterpenes were found from 22.5% in flowers [49] to 48.6% in aerial parts including flowers [46]. Within representative sesquiterpenes in leaves, *α*-zingiberene is the most abundant and was extracted from 5.9% [49] to 14.8% [48], followed by *α*-bisabolol with values ranging from 1.9% [48] to 11.4% [46]. Further ar-curcumene was obtained from 8.3% [46] to 10.6% [48], and *β*-bourbonene showed a presence from a residual 0.1% [48] up to 8.7% [46]. 

The essential oil compositions of the aerial parts (stems and leaves) of *C. albidus* showed only quantitative differences. However, the flowers’ (petals’) essential oil has a different composition, with mainly *α*-zingiberene, *α*-cadinol, ar-curcumene, and *δ*-cadinene [48,49], while the composition of the isolated pollen contained *α*-zingiberene, *δ*-cadinene, and germacrene D within the most abundant compounds [49]. 

Since the analyzed samples come from different places with different climatic and soil conditions, were collected on different dates, and were sometimes analyzed by different methods, comparison of their compositions is only possible to a very limited extent. In addition to these limitations, it must also be taken into account that the species of the *Cistus* subgenus hybridize with each other, which may also have an impact on the composition of the synthesized compounds if species purity has not been ensured beforehand.

#### 5.1.2. Phenylpropanoids from the Essential Oils

Phenylpropanoids are compounds that are built from an aromatic benzene ring and a chain of three carbon atoms and often have hydroxyl and methoxy groups on the aromatic ring. Many phenylpropanoids are natural substances that are formed in plants and microorganisms through the shikimate biosynthetic pathway, with phenylalanine and tyrosine as intermediate compounds [176]. In addition to terpenes, phenylpropanoids are a frequent component of essential oils and represent the majority of natural phenolic substances or their precursors. The phenolpropanoids identified so far in *C. albidus* are eugenol [49,53,177] and chavicol [50].

#### 5.1.3. Diterpenes

As for the diterpenes present in *C. albidus*, these are not usually detected in most analyses, because they belong to the non-volatile terpenes. This is mainly because the analytical methods described are not suitable for detecting non-volatile substances and not because of the absence of these compounds. The diterpenes detected so far are geranyl linalool, geranyl α-terpinene, geranyl p-cymene and 13-epi-mannoyloxide [53], methyl neoabietate [47], 15,16-dinorlabd-8(20)-en-13-one, manool [106], and manoyl oxide [126]. The latter was found in the aerial parts, including the stems, unlike 15,16-dinorlabd-8(20)-en-13-one and manool, which were obtained only from the leaves. 

#### 5.1.4. Tetraterpenes

Tetraterpenes are vital for plant growth, protection against stress, and successful reproduction. So far, lutein, neoxanthin, and zeaxanthin have been identified in *C. albidus* [29].

### 5.2. Phenolic Compounds 

**Figure 5:**
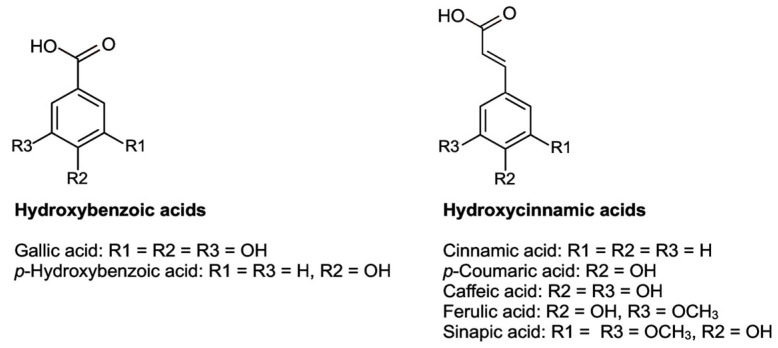
Structures of hydroxybenzoic and hydroxycinnamic acids.

**Figure 6:**
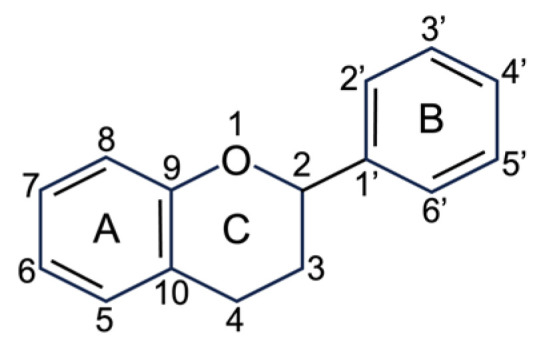
Basic structure of flavonoids with their three characteristic rings which are formed by different biosynthetic pathways. The B ring is derived from the shikimate pathway via phenylalanine.

Phenolic compounds are based on the phenol structure. In general, these compounds can be divided into seven subgroups: simple phenols, hydroxybenzoic acids, hydroxycinnamic acids, coumarins, flavonoids, lignans, and lignins [178]. The concentration of these phenolic substances in plant foods depends, in part, on the plant species, the climate, and the degree of maturity [179]. In the present work, the analytical focus of phenolic compounds lies on the group of hydroxybenzoic acids (basic structure C6-C1), hydroxycinnamic acids (basic structure C6-C3) (Figure 5), and flavonoids (basic structure C6-C3-C6) as they are the most frequent polyphenolic groups present in *C. albidus* samples (Figure 6).

In general terms, the polyphenolic composition of *C. albidus*, listed in Table 2, is very similar to that of other members of the *Cistus* subgenus, such as *C. crispus* and *C. × incanus* (hybrid of *C. albidus × C. criticus*) [5]. A semi-quantitative analysis of the composition of extracts from *C. albidus*, *C. clusii*, *C. ladanifer*, and *C. salviifolius* revealed small differences between them [177] and a series of substances that occur exclusively in *C. albidus*, namely caftaric acid, prunin, and 5-O-caffeoylquinic acid glucoside [12]. 

#### 5.2.1. Flavonoids 

**Figure 7:**
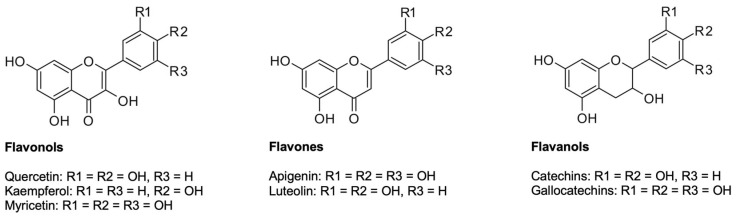
Structure of flavonoids with their three characteristic rings.

Flavonoids contribute to overall fruit color and flavor in plants [178]. In the form of flavones, they are responsible for the yellow hues of the inner petals in the flowers and of the stamens and, in the form of anthocyanidins, for the purple-pink colored petals of *C. albidus*. Flavones often appear as co-pigments of anthocyanins. The interaction of both types of dye explains the simultaneous appearance of yellow and red in different flowers. The flavonoids found so far are flavonols, flavones, flavanols, and tannins (Figure 7). 

**Flavonols and flavones** occur in *C. albidus’* aerial parts as free algycones and glycosides. They are responsible for yellow color nuances and are usually tasteless. Flavonols are distinguished from flavones by the presence of a 3-hydroxyl group (Figure 7). Phenolic substances of the flavonol subclass are present in all plant organs of *C. albidus*. Characteristic representatives of these compounds in *C. albidus* are kaempferol, quercetin, myricetin, myricitrin (myricetin-3-*O*-rhamnoside), and quercitrin (quercetin-3-*O*-rhamnoside) [5]. High contents of myricetin glycoside (7 mg/g dry weight) were detected in *C. albidus* aerial parts, with myricetin-3-*O*-rhamnoside being the primary derivative of myricetin with 83% of total myricetin composition, followed by quercetin glycoside (2 mg/g dry weight), with quercetin-3-*O*-rhamnoside being the most abundant derivative of quercetin, constituting 80% of the total quercetin composition [11]. The only flavones detected so far are diglycosylated apigenin [5] and the isoflavone glycitin 6″-*O*-malonate [12].

**Flavanols** are found predominantly in the leaves of *C. albidus* and contribute particularly to the astringent taste of extracts. Compounds belonging to this subclass have two asymmetrically substituted carbon atoms and can therefore exist as diastereomeric 5,7,3-,4-tetrahydroxyflavanols, catechin and epicatechin. A third hydroxyl group on ring B also results in 5,7,3,4,5-pentahydroxyflavanol gallocatechin, or correspondingly epigallocatechin (Figure 7). Among the polymeric flavanols, the most relevant compounds in *C. albidus* are prodelphinidins such as (epi-)gallocatechins. Procyanidins are other representative flavanols contained in *C. albidus* leaves. Within these oligomeric compounds, (epi-)catechins are most common in *C. albidus* aerial parts [5]. However, combinations of prodelphinidins and procyanidins have also been detected. So far, the following flavan-3-ol compounds have been found: (+)-catechin, (−)-epicatechin, (−)-(epi)gallocatechin, (−)-(epi)gallocatechin-(epi)catechin dimer, (−)-(epi)gallocatechin, and (−)-(epi)gallocatechin-(epi)gallocatein dimer [5,180]. 

**Tannins** are water-soluble, slightly acidic oligomers of polyphenols. They are able to form water-insoluble complexes with protein molecules. In the past, this property of *C. albidus* was used to tan animal skin in leather production [181]. Based on the chemical structure of the monomeric building blocks, tannins present in *C. albidus* could be divided into two groups, the condensed and the hydrolyzable tannins. 

The first group includes the proanthocyanidins (flavanols) already described, which are also known as condensed tannins. They consist of polymerized flavonoid phenols, such as catechins, epicatechin, anthocyanins, and so on. They are correspondingly polymers whose monomeric units consist of phenolic flavans, mostly catechin (flavan-3-ol).

The second group represents hydrolyzable tannins, which are hydrolyzed by the action of acids. These compounds exist as various polyhydroxy compounds, for example as sugar esterified with a phenolic acid [178]. Within this group, hexahydroxydiphenoyl-D-glucose (HHDP-Glc) was detected in *C. albidus* [5]. When the phenolic acid is gallic acid, the compound is called gallotannin. If, on the other hand, esterification with hexahydroxydiphenic acid occurs, this compound is called ellagitannin. Ellagitannins were not found in *C. albidus* aerial parts, except for a residual presence of glucogallin, pedunculagin, and punicalagin gallate [5,11]. 

#### 5.2.2. Phenolic Acids 

Phenolic acids are understood as the hydroxylated derivatives of benzoic acids (hydroxybenzoic acids) and cinnamic acids (hydroxycinnamic acids). Their highest concentration in plants is found in the outer leaves. In *C. albidus* aerial parts, glucogallin [5,11] and gallic acid [5,182] were found. Within the category of phenolic acid derivatives, 5-*O*-caffeoyl quinic acid glucoside, caffeoylquinic glycoside [12], and uralenneoside and rhamnoside of hydroxyferulic acid [5] have also been detected.

**Table 2:** Phenolic compounds identified in *Cistus albidus*. 🌱: aerial parts, including leaves and twigs. n/a: reliable data are not available.

No	Compound	Structure/Class	Presence in	Analytical Reference	Pharmacology
1	Apigenin diglucoside	flavone	🌱	[5]	anticancer, anti-inflammatory, antimicrobial, antioxidant [183]; anxiolytic [184];
2	Caffeic acid	phenolic acid	🌱	[11]	anticancer [185,186,187]; antimutagenic, antihyperglycemic, anti-inflammatory, antioxidant, [188,189]; antmicrobial [186]; cardioprotective [189]; hepatoprotective [190]; neuroprotective [187,191];
3	Caftaric acid	phenolic acid	🌱	[12]	anticancer, antidiabetic, antihypertensive, anti-inflammatory, antimutagenic, antioxidant, hepatoprotective, [192];
4	Cynarin	phenolic acid	🌱	[12]	anticancer, antidiabetic antiulcer, antivirus, antioxidant, hepatoprotective, hypocholesterolemic [193]; cardioprotective [194];
5	(-)-(Epi)catechin	flavanol	🌱	[5,177,180]	neuroprotective [195]; antioxidant [195,196]; antimicrobial [197]; anti-inflammatory, antiallergenic, antivirus, anticancer, skin penetration enhancer, UV-protection [198];
6	(-)-(Epi)gallocatechin		🌱	[5,177,180]	
7	Epigallocatechin-(4*β*-->8)-gallocatechin-(4α-->8)-gallocatechin trimer		🌱	[5,180]	
8	Epigallocatechin-(4*β*-->8)-gallocatechin-(4*α*-->8)-catechin trimer		🌱	[5,180]	
9	(-)-(Epi)catechin-(epi)gallocatechin dimer		🌱	[5]	
10	(-)-(Epi)gallocatechin-(epigallocatechin dimer		🌱	[5]	
11	(-)-(Epi)gallocatechin gallate		🌱	[5]	
12	4,3′,4′-Trimethyl catechin		🌱	[12]	
13	Ferulic acid oligomer	phenolic acid	🌱	[12]	antioxidant [199]; antimicrobial [200]; anti-inflammatory [201]; neuroprotective [202]; antiviral [203]; antiallergic [204]; hepatoprotective [205]; anticancer [206]; antithrombotic [207]; antidiabetic [208];
14	Hydroxy-ferulic acid rhamnoside	phenolic acid glycoside	🌱	[5]	
15	Feruoyl quinic glucoside		🌱	[12]	n/a
16	Gallic acid	phenolic acid	🌱	[5,182]	anti-inflammatory [209,210]; antiobesity, antioxidant [211]; hepatoprotective [210]; anticancer [212]; antifungal [213];
17	Galloyl glucose	flavanol	🌱	[12]	antiviral [214];
18	Glucogallin	flavanol	🌱	[5,11]	antioxidant, anti-inflammatory, antidiabetic, cataract-preventing, antiglaucoma, UV-protective [215];
19	Glycitin 6″-*O*-malonate	isoflavone (flavonoid glycoside)	🌱	[12]	n/a
20	Hexahydroxydiphenoyl- D-glucose	hydrolysable tannin		[5]	antioxidant [216]
21	Kaempferol diglucoside	flavonol	🌱	[5]	antidiabetic [217]; anxiolytic, antidepressant, antiepileptic, anti-inflammatory, neuroprotective, analgesic [218];
22	Kaempferol 3-O-rutinoside		🌱	[5,177]	
23	Luteolin-7-*O*-rutinoside	flavonol	🌱	[12]	antioxidant, anti-inflammatory [219];
24	Myricetin hexoside	flavonol	🌱	[5,177]	anticancer, antidiabetic, antiobesity, cardioprotective, osteoporosis protective, anti-inflammatory, hepatoprotective [220];
25	Myricetin glycoside		🌱	[11]	
26	Myricetin 3-*O*-rutinoside		🌱	[12]	
27	Myricetin-*O*-galloyl-hexoside		🌱	[12]	
28	Myricitrin		🌱	[5]	
29	Oenin	anthocyanin (flavonoid glycoside)	🌱	[12]	anticancer [221]; neuroprotective [222]; anti-inflammatory [223]; antioxidant [224];
30	Pedunculagin	ellagitannin	🌱	[5,11]	antioxidant, anti-inflammatory, dermatoprotective [225]; anticancer [226]; antibacterial [227];
31	Pelargonidin 3-*O*-(6″malonylglucoside)	anthocyanin (flavonoid glycoside)	🌱	[12]	cardioprotective, neuroprotective [228,229];
32	Peonidin 3-*O*-(6″-p-coumaroyl) glucoside	anthocyanin (flavonoid glycoside)	🌱	[12]	cardioprotective, neuroprotective [228,229];
33	Petunidin	anthocyanidin (O-methylated flavonoid)	🌱	[12]	antioxidant [230]; cardioprotective, neuroprotective [228,229];
34	Procyanidin	flavanol	🌱	[12]	antioxidant [231]; antibacterial [232]; anticancer [233]; anti-inflammatory [231,234];
35	Prunin	flavanone	🌱	[12]	antihyperlipidemic, antihyperglycemic, antidiabetic [235];
36	Punicalagin	ellagitannin	🌱	[11]	anti-inflammatory, antioxidant, neuroprotective, hepatoprotective, cardioprotective, antiviral, antimicrobial, anticancer, antidiabetic, antihyperlipidemic, gastroprotective [236];
37	Punicalagin gallate		🌱	[11]	
38	Quercetin	flavonol	🌱	[12]	anticancer, antitumor, anti-inflammatory, antiviral, antihypercholesterolemia, antihyperglycemic, antioxidant, antibacterial, cardioprotective, gastroprotective, hepatoprotective, antihypertensive, nephroprotective, neuroprotective [237]; antidepressant [238]; antimicrobial [239], antifungal [240]; antiallergic [241] antiobesity [242];
39	Quercetin glucoside		🌱		
40	Quercetin 3-*O*-glucoside		🌱	[12]	
41	Quercetin 3-*O*-(2’-cumaroyl)- rutinoside		🌱	[12]	
42	Quercetin 3,4-diglucoside		🌱	[12]	
43	Quercetin 3-*O*-(2’-caffeoyl)-rutinoside		🌱	[5]	
44	Quercitrin		🌱	[5,11]	
45	Rutin		🌱	[5,11,12,177]	
46	Quinic acid	phenolic acid	🌱	[12,177]	radioprotective [243]; neuroprotective [244]; anti-inflammatory [245]; 5antiviral [246];
47	3*p*-Coumaroylquinic acid		🌱	[12]	n/a
48	3-Caffeoylquinic acid		🌱	[11]	antioxidant, anti-inflammatory [247];
49	Caffeoylquinic glycoside	phenolic acid glycoside	🌱	[12]	enzyme inhibition, hepatoprotective [248];
50	Isorhamnetin- *O*-rutinoside	flavonol	🌱	[5]	anti-atherosclerosis, cardioprotective, neuroprotective, anticancer, antihypertensive, antioxidant antihyperglycemic, hepatoprotective, anti-inflammatory, anti-osteoporosis, antiobesity, UV-protection [249]; antimicrobial [250];
51	3-*O*-Methylrosmarinic acid	caffeic acid ester	🌱	[12]	anti-inflammatory, antioxidant, antidiabetes, antivirus, antitumor, neuroprotective, hepatoprotective [251];
52	Methoxy dihydroferuoyl methyl rosmarinic acid		🌱	[12]	
53	Dihydroxy-dihydro feruoyl methyl rosmarinic acid		🌱	[12]	
54	*p*-Hydroxy benzil rosmarinic acid		🌱	[12]	
55	Shikimic acid dimer	phenolic acid	🌱	[12]	anticoagulant [252]; antithrombotic [253];
56	6′-O-Sinapoyl sucrose	hydroxy-cinnamate sucrose esters	🌱	[12]	antioxidant [254];
57	Syringyl shikimic acid dimer	phenolic acid	🌱	[12]	n/a
58	Uralenneoside	p-hydroxy- benzoic acid alkyl ester	🌱	[5]	n/a

### 5.3. Carbonylic Compounds 

Aldehydes are odoriferous aromatic substances in plants. These often arise from substances containing linolenic acid during harvesting, crushing, or preparation [255]. The aliphatic aldehydes, octanal [49], nonanal, and decanal [49,53], were exclusively identified in pollen and may be responsible for the typical sweet smell of the flowers [256]. On the other hand, tetradecanal, undecanal, and dodecanal were present in the aerial parts [53]. These compounds may contribute to the typical sweetish odor of *C. albidus* with its undertone of oranges, lemons, and roses [256,257].

### 5.4. Phytohormones and Vitamin E

Tocopherols and tocotrienols are also present in leaves and seeds. More than 75% of the vitamin E present in the seeds was in the form of *α*-tocopherol, followed by *α*-tocotrienol and *γ*-tocopherol [29,35,117], which is the immediate precursor of *α*-tocopherol. Phytohormone and vitamin studies further revealed the presence of the jasmonates 12-oxo-phytodienoic acid (OPDA), jasmonic acid (JA), and jasmonoyl-isoleucine (JA-Ile) and plastochormanol-8 [20], carotenoids, and abscisic acid, with *α*-tocopherol being the most abundant [27]. A negative correlation was revealed between vitamin E and OPDA accumulation in *C. albidus* under winter conditions, while a positive correlation was found between JA and *α*-tocopherol [20]. A significant positive correlation was also detected between hydration and total leaf chlorophylls due to the protection mechanism of tocopherols from the photosynthetic apparatus. Therefore, higher levels of *α*-tocopherol were observed under abiotic stress conditions and when the leaves showed an orientation more perpendicular to the solar rays [20,27,28,258]. 

Drought stress can induce an increase in the concentrations of abscisic acid and H_2_O_2_ in the leaves, inducing an increase in ascorbic acid, maintaining and even decreasing the oxidative state of ascorbate, thus protecting plants from oxidative damage [30,259]. In addition, cytokinins that act as nitric oxide scavengers and are involved in the modulation of the abscisic acid response have been reported [29]. 

*C. albidus* seeds obtained from mature plants showed higher concentrations of *α*-tocopherol, JA, and salicylic acid than those obtained from younger plants. Auxin (indole-3-acetic acid) content was also significantly higher in seeds from older plants. Gibberellic acid GA4 and its precursor GA24 were also found in seeds [27]. No differences were detected between the concentration of cytokinins in seeds from older and younger plants, except for zeatin, which was significantly higher in seeds from older plants. Zeatin was the main form of cytokinin found in the seeds of *C. albidus* [27].

### 5.5. Alkanes 

Among the volatile compounds isolated from aerial parts of *C. albidus*, tricosan [53], tetracosan [47], pentacosan [43], octacosan [47], and docosan [43,47] were found. n-Tridecane was present in petals, and n-tetradecane was identified in both petals and pollen, while n-hexadecane was detected only in pollen, and n-pentadecane was found in leaves [43,47,53].

### 5.6. Other Compounds: Fatty and Carboxylic Acids

Although fatty acids are products of the primary metabolism, they are described here for the role they may play in the bioavailability of pharmacologically active compounds, as described below. Among the fatty acids found in the aerial parts of *C. albidus* are tetradecanoic, primaric and pentadecanoic acid [47], nonanoic acid [106], palmitic acid [53], and butanoic acid [49]. The fatty acid composition of the seeds showed significantly higher levels of polyunsaturated fatty acids as well as very-long-chain saturated fatty acids for older plants due to their higher levels of linoleic acid [27]. In addition, the carboxylic acids methacrylic acid [106] and quinic acid [12,177] were identified.

## 6. Preparation Methods of the *C. albidus* Extracts 

Studies of the pharmacological properties of traditional medicines based on *C. albidus* preparations are related to the presence of terpenoids and polyphenols. In order to understand the use that has been given to *C. albidus* traditionally, it is necessary to first review the preparation methods used in popular medicine of this plant due to their influence on the pharmacological effect.

### 6.1. Traditional Preparations

For the different applications, traditionally, only the aerial parts of *C. albidus* were harvested, mainly the leaves, but also flowers, flower buds, and to a lesser extent stems. The traditional preparation of *C. albidus* varies from an infusion to a prolonged decoction, while the dose usually used is around 3 g per 100 mL of water, taking a cup (150 mL) two or three times a day [10].

Within traditional preparations, decoction is the most used technique. It consists of boiling the plant material for a certain period of time and letting it rest afterwards. This method is primarily suitable for thermostable and water-soluble phytochemicals. During decoction, several compounds undergo chemical modifications. For example, catechins undergo epimerization, which is a change in their configuration relative to one of their stereogenic centres. Epimers, specifically epicatechins and epigallocatechins, have been shown to have important health benefits. It has been found that this epimerization occurs more readily in water with alkaline pH values than in purified water [260]. In addition, it has been shown that at temperatures greater than 98 °C, epimerization occurs faster than its degradation [261], so it can be deduced that the traditional preparation of *C. albidus* is the most effective way to extract catechins and their epimers. However, for green tea, the levels of epicatechin, epicatechin gallate, epigallocatechin, and epigallocatechin gallate were reported to increase only during the first 3 to 5 min of preparation (infusion at 85 °C), and the proportion of these flavonoids decreased as time increased. In contrast, another study found that levels of catechin, gallocatechin, and gallocatechin gallate increased continuously with the length of preparation time [262]. Taking these results into account, the pharmacological activities referred to in traditional use could be optimized by limiting the decoction time. Nonetheless, thermolabile compounds are lost in the decoction process. As a result, monoterpenes should not be contained in the resulting extract. Sesquiterpenes, however, would not be affected by extracting temperatures around 100 degrees but by low solubility in water due to their lipophilic character. It can therefore be assumed that terpenes play a minor role in the traditional decoction of plant material.

On the other side, probably in order to use the entire compound spectrum of the plant, based on both terpenes and polyphenols, the dried and crushed leaves sometimes were used directly (orally) [9,263,264,265,266]. This usage ensures that the resulting medicine is rich in polyphenols terpenes and other volatiles. However, a loss of several terpenes and oxidative reactions could be induced by the drying process, as reported for other species as *Cannabis sativa* [267,268]. 

### 6.2. Actual and Alternative Extraction Methods

In general, solid liquid extraction is the most commonly used method for extractive purposes. When using water as a solvent, the extraction is carried out at temperatures ranging between 20 and 50 °C. For extraction with solvents such as ethanol, methanol, and acetone, temperatures commonly used range from 60 to 80 °C [269]. For temperatures above 80 °C, a decrease in the yield of total polyphenols and proanthocyanidins has been reported, suggesting that it is due to their degradation; this process accelerates with temperatures above 70–80 °C [270,271]. However, other studies have shown that the higher the temperature, the more efficient the extraction is, since the heat makes the cell walls permeable, increasing the solubility and the diffusion coefficients of the compounds to be extracted and decreasing the viscosity of the solvent [272]. The extraction temperature has a significant effect on the types of polyphenols that are extracted. However, the thermal degradation of different types of polyphenols varies according to the pre-treatment, type of solvent, pH, and time used [269].

On the other hand, a mixture of polar and nonpolar solvents should result in an efficient extraction of both polyphenols and terpenes at the same time. However, with a few exceptions, in its traditional use, *C. albidus* has not been extracted by solvents with combined properties, such as EtOH. Studies have revealed low efficiency of these extraction methods, as well as environmental problems due to the requirement of large volumes of solvents, especially if they are organic [273]. Furthermore, traditional extraction processes often involve an intermediate step before extract concentration, which is a time- and energy-consuming process. 

The compounds of *C. albidus* listed in this review were obtained from the aerial parts of the plant by means of distillation or extraction (water, ethanol, chloroform, pentene). Essential oils were isolated from fresh aerial parts by hydro-distillation using a Clevenger’s apparatus for between 2 h and 8 h. The isolated oil was dried over anhydrous sodium sulphate, and the essential oils were stored at +4 °C, in glass vials at dark, until analyses [46,48,49,126]. Pentane extracts [47] and a mixture of pentane/diethyl oxide [53] were also used to isolate essential oils. 

To analyze the polyphenols of *C. albidus*, aerial parts were usually air dried and ground into powder to a size between 2 and 5 mm and then extracted by ethanol maceration (1:4, *w*/*v*) up to 72 h or acidified (0.1% HCl) methanol (1:3, *w*/*v*) for 24 h. The plant extracts were filtered or decanted (<24 h) and dried under vacuum using a rotary evaporator at temperatures ≤ 50 °C. Extracts were stored in the dark between 2 and 8 °C until used for the assessment [12,177,274]. 

A special case of polyphenol extraction is the flavonol-enriched *C. albidus* chloroform extract described by Tahiri et al. [13]. Here, the supernatant of the ethanol extract was further partitioned in ethyl acetate and water (1:3:1, *w*/*v*/*v*) and then separated into an organic and an aqueous phase. The organic phase of ethyl acetate was further partitioned in chloroform and water (1:3:1, *w*/*v*/*v*), yielding an organic and an aqueous phase of chloroform, respectively.

For the aqueous extracts, fresh aerial parts were ground to a maximum size of 5 mm and macerated for 2 h with distilled water (1:5–20, *w*/*v*) at temperatures between 60 and 90 °C. The filtered extract was then centrifuged and concentrated by rotatory evaporation and kept at 4 °C until use [5,11,177,182].

Alternative methods include supercritical CO_2_ extraction, microwave-assisted extraction, ultrasound-assisted extraction, enzyme-assisted extraction, pressurized fluid extraction, or a combination of these approaches. These methods require less solvent volume and extraction time, while at the same time producing higher yields and reducing toxic residues. Although these methods have not yet been described with *C. albidus*, they may be an interesting alternative that should be further explored, as has been done for other vegetal matrices from *Rosmarinus officinalis* [275] and *Salvia miltiorrhiza* [276].

## 7. Bioavailability of the Groups of Compounds Found in the *C. albidus* Extracts

Bioavailability is a pharmacokinetic concept that refers to the proportion and the pace at which a served dose of a drug reaches its therapeutic target, considering the tissue on which it acts. According to this definition, this term includes the absorption, metabolism, and distribution of a single compound. In general, secondary plant substances are absorbed, distributed, and metabolized in a similar way to xenobiotics [176]. 

Despite the fact that it has not yet been proven that extracts from *C. albidus* will be bioavailable, in this subsection, an attempt will be made to derive the bioavailability theoretically.

The structure of flavonoids affects their solubility in water, which in turn affects their absorption and bioavailability. Flavonoids with a more hydrophobic structure. such as flavonols, may have lower bioavailability compared to those that are more hydrophilic, such as flavanols. Flavonols contain a high proportion of hydrophobic groups, such as aromatic rings and aliphatic chains, which make them less soluble in water and more soluble in lipids [178]. Flavonoids with a more hydrophobic structure present in *C. albidus* are apigenin, quercetin, myricetin, and kaempferol. They must undergo metabolism in the gut to be absorbed [277]. 

On the other hand, the bioavailability of flavanols and condensed tannins is related to their catechol ring structure with hydroxyl groups that can form hydrogen bonds with water molecules, which makes them more soluble in water and more readily absorbed by the body. They cannot be hydrolyzed in the stomach and are therefore broken down into phenolic acids by microorganisms in the large intestine. The latter are then resorbed in the large intestine [277]. Thus, flavanols, such as catechins, are relatively soluble in water and can be absorbed by the body through passive diffusion in the small intestine [278]. 

The gut microbiota plays a crucial role in the metabolism and absorption of flavonoids by breaking them down into smaller metabolites that can be absorbed and utilized by the body. Flavonoids are not readily absorbed in their native form and must therefore undergo extensive metabolism in the gut to be absorbed and utilized [277]. The gut microbiota contains a diverse community of microorganisms, including bacteria, fungi, and viruses, that can metabolize flavonoids through various pathways. Some bacterial species have specialized enzymes that can break down flavonoids into smaller metabolites that are more bioavailable, such as phenolic acids and aromatic compounds [278,279].

Phenolic acids are generally more water-soluble than flavonoids and can be absorbed by the body through passive diffusion in the small intestine. Overall, the bioavailability of unesterified phenolic acids is high. These can be absorbed quickly in the small intestine without prior hydrolysis. However, phenolic acids are mainly present in esterified form, so that due to the lack of esterases in the human digestive tract, there is no absorption in the small intestine. Esterified phenolic acids are therefore first hydrolyzed by microorganisms in the large intestine, and the resulting metabolites are then resorbed [278].

Ellagitannins found in *C. albidus* are considered to have relatively low bioavailability. This is because they are highly complex molecules that require microbial metabolism in the colon to be broken down into smaller, absorbable metabolites [278].

Regarding the terpenes and other poorly absorbed compounds, it is said that both their low permeability across absorption barriers and reduced solubility in biological fluids decreases their bioavailability [178]. However, it might be increased through the natural presence in *C. albidus* of certain unsaturated fatty acids by enhancing micellarization during the digestion process. [280]. These compounds might increase the absorption of terpenoids and polyphenols present in *C. albidus* samples. 

Furthermore, phenolic and terpenoid compounds, in the presence of *α*-tocopherol, have been shown to promote transport through the blood brain barrier (BBB) [281]. Moreover, studies have shown that borneol could increase the permeability of BBB by significantly losing the intercellular tight junctions (TJ) and increasing the number and volume of fluid endocytosis (pinocytosis) in in vitro models, thus enhancing the bioavailability of drugs [282,283,284,285]. Thus, several compounds of *C. albidus* may induce potential mechanisms resulting in an increased bioavailability. Furthermore, the absorption and bioavailability of *C. albidus’* compounds can be influenced by other factors such as food matrix, processing (extraction), and individual variations.

## 8. Therapeutical Uses

### 8.1. Traditional Uses

Plant resources have always been an integral part of human society throughout history. Until the middle of the last century, traditional medicines provided an alternative and inexpensive source of primary health care for the rural population. However, with access to synthetic drugs, a large number of medicinal plants became obsolete, the memory of which in the population, after only two generations, is being lost. 

One of these medicinal plants is *C. albidus*, which has been used in traditional folk medicine for a variety of illnesses [10,286,287,288], especially for the treatment of fever, diarrhea and other gastrointestinal illnesses [8], skin diseases, rheumatism, and various inflammatory diseases [182]. For the sake of completeness, it is mentioned here that *C. albidus* has also been used as a tanning agent [181], as an insect repellent, and as a substitute for tobacco, highly appreciated, moreover, for its hypotensive effect [49,288,289].

The decoction of leaves was traditionally used in the Spanish Levant as a tranquilizer, in the Baixa Plana as a sedative [10], and as a remedy against Parkinson’s symptoms in Mallorca [290,291]. To relieve toothache, mouthwashes were made with a decoction of its leaves and flowers. A sip of the resulting liquid, once cold, was kept in the mouth for some time [266,292,293,294]. In addition, the decoction of the aerial parts was used as an external antiseptic, for wounds and skin infections [293,294,295,296]. In the Spanish Basque Country, several uses were reported. For example, decoction was applied for the treatment of ulcers and for the treatment of gangrene, and fresh leaves were used directly on the wound for disinfection [297].

In the Mediterranean region, the decoction of the aerial parts (leaves, stems, and flowers) has been used to regulate blood pressure [298,299]. It has also been a frequent remedy for hemorrhoids and to treat bruises and varicose veins [290]. The decoction of flowers and leaves has also been popularly used as an analgesic for oral infections [293] and for hepatoprotection in Granada and Mallorca [290,298]. The decoction of the fresh aerial parts, including the flowers, was used as a remedy against colds and flu infections, and against bronchitis [9,290,299] and whooping cough [10]. In the Spanish peninsula, *C. albidus* decoction has also been used as a remedy for osteoarthritis in the province of Jaen [266] and for rheumatism in the Valencian community and the Province of Jaen [292,300]. In addition, it was used as an external antiseptic for wound healing and skin infections in the provinces of Castellon, Mallorca, and Almería [10,290,293], and in Morocco [263,265]. In Sardinia (Italy), a traditional use is reported in poultices and ointments, which were applied directly to the wound [301].

In cases of gastrointestinal infections, in Almería (Spain), an infusion of dried leaves was prepared to reduce abdominal pain [293,298]. Against colic, in Castilla-La Mancha and Murcia (Spain) an infusion of young and tender shoots was administered, but it was also supplied by oral ingestion of the powder of dry leaves for treatment [9]. The dried leaf powder also served as an antidiarrheal in Jaen [266].

Infusions of fresh flowers and leaves have been used as an antiseptic for the urinary tract in Murcia [302] and also as an anti-inflammatory for orchitis in Valencia [303].

### 8.2. Scientific Evidence Confirming Traditional Uses

#### 8.2.1. Antimicrobial Activity

The antimicrobial activity of *C. albidus* is attributed to two main compound classes, terpenes and polyphenols. Studies have shown that both terpenes and polyphenols have potent antibacterial and antifungal activities [12,46,177,274]. However, there are few studies on the antimicrobial activity of *C. albidus*. 

The terpenoid fraction of *C. albidus* extracts has been found to exhibit antimicrobial activity against *S. aureus, Bacillus subtilis, Listeria monocytogenes, Klebsiella pneumoniae*, and *Candida albicans* [46]. Gram-positive bacteria are more affected by terpenoids than gram-negative bacteria [46]. The inhibitory activity against *B. subtilis, S. aureus, L. monocytogenes, K. pneumoniae*, and *C. albicans* is seen at a minimum inhibitory concentration (MIC_50_) of 20 µ/mL, while *E. faecalis, E. coli*, and *E. freudii* are not affected [46].

The polyphenolic compounds, such as gallic acid and some glycosylated derivatives of myricetin and quercetin, are found in *C. albidus* extracts that exhibit strong antimicrobial activity against *S. aureus* [46,177]. However, the specific role of polyphenols in the antimicrobial capacity of *C. albidus* extracts against several bacteria, including *S. aureus* and *E. coli*, is not yet clear. Some studies suggest a positive correlation between higher content of polyphenols and higher antibacterial activity against both Gram-positive and Gram-negative bacteria [177]. The antimicrobial activity may be attributed to a synergistic effect of different compounds, including phenolic and terpenoid. The polar fraction of *C. albidus* extracts, including the butanol extract, the spray-dried aqueous extract, and the ethyl acetate extract, all exhibit strong antimicrobial activity against *S. aureus* with MIC_50_ of 2.5 mg/mL [12], 60.0 µg/mL [177], and 1.25 mg/mL [12], respectively. The hydroalcoholic extract of *C. albidus* also shows strong activity against *E. coli* with MIC_50_ of 233 µg/mL [177].

#### 8.2.2. Anti-Inflammatory, Antinociceptive, Analgesic, and Sedative Activity

The anti-inflammatory, antinociceptive, analgesic, and sedative effects reported in the traditional use of this species are probably based on both terpenes and polyphenols and may be the result of a synergy of both groups of compounds, each with different effects on the central nervous system (CNS). Among single compounds of the terpenoid class present in *C. albidus* with a reported neurophysiological activity are the monoterpenes bicyclogermacrene, borneol, *p*-cymene, germacrene-D, linalool, myrcene, α-phellandrene, safranal, and thymol and the sesquiterpenes abscisic acid, *⍺*-bisabolol, *β*-caryophyllene, caryophyllenol, guaiol, selin-11-en-4-*α*-ol, *β*-sesquiphellandrene, and α-zingiberene (Table 1). 

A recent study with α-zingiberene, one of the principal compounds in *C. albidus*, showed a significant anti-inflammatory activity resulting in a reduction in angiogenesis, macrophage activation, and the activity of metalloproteinases [165]. In a mice model, α-zingiberene was found to reduce neuroinflammation through histone deacetylase 1 (HDAC1) inhibition. Neuropathic pain results from microglia-spinal overexpression of HDAC1, and α-zingiberene was found to be a promising HDAC1 inhibitor, with an IC_50_ of 2.3 ± 0.1 µM. Further, the administration of α-zingiberene reduced thermal hyperalgesia and mechanical allodynia [163]. Moreover, germacrene-D, another principal compound, was shown to have the potential for the development of pharmaceutical formulations with anti-inflammatory, antinociceptive, and analgesic activities in non-toxic concentrations [144,304].

Due to the polyphenolic composition, significant anti-inflammatory and antinociceptive activities were observed in *C. albidus* extracts, especially for several flavonols [13,305]. Anxiolytic and analgesic properties have also been observed for apigenin diglucoside [184], which selectively binds with high affinity to GABA_A_ receptors. Similarly, kaempferol, myricetin, and quercetin derivatives are potentially responsible for anxiolytic, antinociceptive, and anti-inflammatory activity without exerting psycho-modulatory effects [13,184,306,307,308]. In addition, quercetin and kaempferol exhibited antidepressant effects. It has been demonstrated in several studies that both compounds act as monoamine oxidase inhibitors [238,309].

In a murine model, a flavonol-enriched *C. albidus* extract exhibited a substantial reduction in paw oedema and significantly inhibited nitrite generation without affecting the cell viability of lipopolysaccharide-stimulated murine peritoneal macrophages [13]. The same study observed a downregulation of the proinflammatory enzymes cyclooxygenase (COX-2) and inducible nitric oxide synthase (iNOS) in macrophages treated with the *C. albidus* extract, as well as a decrease in *p*38 mitogen-activated protein kinases (MAPK) phosphorylation. Furthermore, a high antinociceptive activity was observed at a concentration of 100 mg/kg and proved to be as efficient as acetylsalicylic acid at concentrations of 200 mg/kg. An HPLC-DAD-ESI-MS/MS analysis of the used chloroform extract revealed that kaempferol and quercetin derivatives were potentially responsible for such effects [13]. It is suggested that these compounds may act through a CNS-mediated analgesic mechanism. Both effects suggest that the polyphenolic composition of this species may act on the central and peripheral nervous system through inhibition of the mechanisms of pain, acting as a sedative [13,310].

## 9. Potential Pharmacological Applications of *C. albidus* and Their Mechanisms

The pharmacological activity of traditional preparations of *C. albidus* is mainly based on the antimicrobial, anti-inflammatory, antinociceptive, and analgesic effects of several of its compounds. Most of these activities have been confirmed by recent studies as described in previous sections, but taking into account the whole spectrum of bioactive substances included in *C. albidus* samples, new applications derived from the above-mentioned pharmacological activities can be suggested, opening the field for future research and uses. In this context, labdane-type diterpenes are promising compounds that have been shown to exert strong antiviral activity. For example, manoyl oxide showed a high capacity to suppress the dengue virus. In a fraction of labdanum diethyl ether extract at a concentration of 31.25 μg/mL from *C. creticus*, the proliferation of this virus was suppressed by 100% [311].

In the field of cancer research, evidence suggests that 13-epi-manoyl oxide, a characteristic labdane-type diterpene of *C. albidus,* may play a pivotal role in antitumor activity, inducing apoptosis in leukemic cell lines through multiple pathways [170,312,313,314]. Flavanols such as prodelphinidins and epigallocatechin-gallocatechin-catechin oligomers were also shown to exert a significant growth-inhibitory activity against human prostate cell lines by blocking cell cycle partly at the G1/G0 phase and activating caspase-3, as was shown for prodelphinidins extracted from *C. albidus* [14], synthetic prodelphinidins [315], and oligomeric proanthocyanidins from *Vigna angularis* [316]. The cytotoxic effect was suggested to be related with the presence of the pyrogallol moiety [14]. However, due to the composition of *C. albidus*, future applications could focus on the prevention of neurodegenerative diseases.

### 9.1. Prevention of Neurodegenerative Diseases

A potential field of application regarding phenolic compounds from *C. albidus* is the prevention of neurodegenerative diseases (NDDs). The potential application of the phenolic compounds of *C. albidus* for NDD prevention is suggested based on a combination of properties such as free radical scavenging and heavy metal chelation.

#### 9.1.1. Free Radical Scavenging

Pathogenesis of NDD is influenced by oxidative stress [317]. Studies have shown the preventive effects of antioxidants in this regard, and in particular, an improvement in cognitive and neural functions was observed [318]. In addition, human CNS studies found that antioxidants can improve cerebral blood flow (CBF) [319,320]. This effect is particularly significant since it influences the maintenance of cognitive performance through adult neurogenesis [321] by improving vascularity [322]. *C. albidus* is rich in antioxidants of both terpenes and polyphenols—especially the phenolic content, with high amounts of flavonols, phenolic acids, flavanols, anthocyanins, and tannins. However, although phenolic compounds are the most abundant and potent antioxidants in *C. albidus*, there are also non-phenolic compounds, such as terpenes, that contribute to the overall antioxidant activity of this species.

Within polyphenols, the flavonols myricetin and quercetin, both a hydroxylated form of kaempferol, are capable of inducing the enzyme glutathione-S-transferase, which is involved in resistance to oxidative stress. Myricetin and quercetin and their glycosides, especially the rhamnosides, act directly as free radical scavengers [323]. They are capable of preventing DNA, protein, and membrane damage due to their aromatic hydroxyl groups [324]. In particular, quercetin acts as a potent radical scavenger based on three structural parts—an *O*-dihydroxy structure (catechol in ring B), a 2,3-double bond in combination with a 4-oxo group, and the additional presence of a 3- and 5-hydroxyl group [178].

Furthermore, it was demonstrated that flavanols such as epicatechins and phenolic acids such as gallic acid exert strong neuroprotective effects. They are able to block the neurotoxic effects caused by oxidized proteins. For example, some HIV proteins are known to cause neurotoxicity in humans through mechanisms that activate macrophages and glial cells, inducing the production of oxidative stress. Epicatechins have been shown to neutralize this effect [325]. In another study, prior to gamma radiation exposure, epicatechin treatment prevented liver and testicular damage due to oxidative stress produced by free radical formation as a result of radiation [326], suggesting that epicatechin may present protective effects in patients undergoing radiotherapy. It was further shown that epicatechins decrease the susceptibility of low-density lipoproteins to oxidation, thus preventing the onset of atherosclerosis [327]. In addition, neuroinflammation in a PD-induced mouse model was significantly reduced when animals were given gallic acid (100 mg/kg) by attenuating heme oxygenase-1 (a redox-regulated protein) and α-synuclein aggregation (an indicator of neurodegeneration of the CNS), suggesting that gallic acid is capable of inhibiting lipopolysaccharide-induced oxidative stress and protein conjugation [328].

In general, several studies have demonstrated a positive correlation between the total phenolic content of *C. albidus* and its antioxidant activity [12,20,30,182,259]. Bouyahya et al. [274] determined the total phenolic content for an ethanolic extract of *C. albidus* as high as 112.48 ± 1.78 mg gallic acid equivalents per gram extract. The antioxidant activity (2,2-diphenyl-1-picrylhydrazyl radical scavenging assay, DDPH) was determined as well, with high values between 27.26 and 142 mg Trolox equivalents per gram dry weight by Gonçalves et al. [182] and Lukas et al. [11], respectively. A study of *C. albidus* from Sardinia showed the ability to scavenge DPPH radicals from *C. albidus* extracts with EC_50_ values of 31.93 μg mL^−1^ for the butanol fraction, 22.23 μg mL^−1^ for the ethyl acetate fraction, and 33.24 μg mL^−1^ for water [12]. 

Regarding the antioxidant activity of terpenes in *C. albidus*, these compounds are present in smaller but not negligible quantities and may be responsible for a synergistic effect. They may improve symptoms caused by inflammation processes, inhibiting them in different steps of the inflammatory process, as demonstrated by a series of studies [130,306,329,330]. The terpenes present in *C. albidus* with potential activity on oxidative stress are *α*-bisabolol, *α*-pinene, *α*-zingiberene, *β*-caryophyllene, *δ*-limonene, myrcene, p-cymene, linalool, and humulene [331]. The mechanisms of action involved in the antioxidant activity are mainly the reduction of lipid peroxidation induced by H_2_O_2_, decreasing the formation of ROS and the release of NO. Furthermore, terpenes can increase catalase, superoxide dismutase, and peroxidase activities; reduce glutathione content; and restore the mitochondrial membrane [332]. On the other hand, α-pinene, limonene, and *β*-caryophyllene are GABA receptor agonists, decreasing the activities of acetylcholinesterase and lipoxygenase. They can arrest the cell cycle in the G2/M phase [332].

It has been claimed that α-bisabolol exhibits antioxidant activity in chemical and/or biological assays. Studies have indicated that α-bisabolol significantly inhibits luminol-amplified chemiluminescence at concentrations ranging from 7.7 to 31 μg/mL for *Candida albicans* and N-formyl-methionyl-leucyl-phenylalanine, respectively. A similar effect was observed in the SIN-1 and H_2_O_2_/HOCl systems, suggesting that *α*-bisabolol is a means of enhancing antioxidant capacity [333].

Limonene, one of the most abundant terpenes in nature, was found to exert antidepressant-like effects in mice caused by reduced nitrite levels in the hippocampus [74]. In addition, it has been shown that limonene reduced the inflammatory response and decreased the levels of inflammatory cytokines such as IL-1, IL-6, and TNF-α, which are related to depressive symptoms [334].

In relation to linalool, there are several in vitro and in vivo studies that show strong anti-inflammatory and antioxidant activity, which confers it with important neuroprotective activity [335,336]. Its mechanism of action consists in reducing the activation of NF-*κ*B and preventing its nuclear translocation [337].

#### 9.1.2. Heavy Metal Chelation

Another factor associated with ROS is the accumulation of heavy metals in the CNS, which can be treated with chelation. These metals are essential for biological functions in plants and animals, but their chemical properties can cause toxicity by interfering with homeostasis. They may bind to inappropriate protein sites, displacing natural binding sites and causing cellular malfunction. Heavy metals have been found to cause oxidative damage to biological macromolecules, primarily by binding to DNA and nuclear proteins. Exposure to heavy metals is related to proinflammatory cytokines resulting in neuronal damage through neuroinflammation and can lead to various disorders and diseases [338]. 

Numerous studies have demonstrated a connection between AD, PD, and dementias, and an exposure to heavy metals due to oxidative stress caused by the formation of free radicals [339,340]. Results from several studies even suggest associations between the levels of metals during pregnancy and autism spectrum disorder (ASD) in children [341,342,343]. In a recent study, arsenic, cadmium, copper, mercury, manganese, magnesium, and lead were identified in the development of ASD. The results of this study suggest that the impact of these metals may be related, in addition, with attention deficit hyperactivity disorder (ADHD), sharing neurochemical and neurodevelopmental pathways [344].

Chelation is a process in which a molecule, known as a chelating agent, forms a complex with a metal ion by surrounding it and holding it in a stable, water-soluble form. Chelation can occur through the formation of coordinate covalent bonds between the metal ion and the chelating agent [345]. The metal chelation potential of polyphenols is highly dependent on catechol moieties and combinations of hydroxyl and carbonyl groups [346]. Combinations of these groups and moieties define metal-binding sites. Chelating agents are compounds that bind to metal ions and prevent them from participating in reactions that can generate reactive oxygen species (ROS). Metal ions can catalyze the production of ROS by participating in reactions that generate highly reactive oxygen species. By binding to these metal ions, chelating agents can prevent them from participating in these reactions, reducing the amount of ROS that are produced. Several polyphenols present in *C. albidus* may exert both antioxidant and chelating properties, as was shown for isolated polyphenols [347,348], and can form stable five- or six-membered rings with oxophilic transition metal ions such as iron, copper, and zinc, which are abundant in the CNS [348,349]. 

Flavonoids such as kaempferol, quercetin, myricetin, and luteolin, as well as flavanols such as catechins have multiple oxygen-containing functional groups, such as hydroxyl (-OH) and carboxyl (-COOH) groups, which are capable of binding to metal ions and forming chelates [348]. The chelating effect of these compounds may also be enhanced by their ability to form hydrogen bonds with metal ions, which further stabilizes the complex [350].

There is evidence that the free-radical-scavenging and metal-chelating effects of aqueous extracts of *C. albidus* could contribute to the prevention of Fe^2+^-induced lipid peroxidation [182]. Infusions of *C. albidus* showed a high capacity to form complexes with Fe^2+^ of 66.63% at a concentration of 1.6 mg mL^−1^. This is especially noteworthy because Fe^2+^ chelation was suggested to be a more important mechanism than direct free radical scavenging for the prevention of lipid peroxidation [182].

## 10. Toxicity

Aside from the fact that this species has been used as an insect repellent and its dead leaves are allelopathic to other plant species, to date, no intoxication produced by *C. albidus* has been reported for mammals. Studies to evaluate the toxicity of *C. albidus* extracts have not yet been performed.

For the genus *Cistus,* in general, there is no evidence of toxicity. However, isolated cases of intoxication in bovines have been associated with a possible ingestion of *C. salvifolius* [351,352]. However, the responsible substances contained in some species of the genus *Cistus*, especially in *C. salvifolius*, which could cause toxic effects, are not yet clear [353] and may be related to their tannins. Tannins differ from other phenolic compounds by their ability to precipitate proteins, exerting a direct inhibition of digestive enzymes that leads to the formation of indigestible complexes for ruminants. Bacterial fermentation converts tannins into gallic acid and pyrogallol, both of which are nephrotoxic to cattle [351]. It is noteworthy that *C. albidus* was widely used in ethno-veterinary medicine for a variety of diseases [9,299,354]. For example, a decoction of leaves and flowers of *C. albidus* was popularly used to treat rumination syndrome in horses [9].

## 11. Conclusions

The composition of secondary metabolites of *C. albidus*, with more than 200 identified compounds, supports the widespread use of this species in folk medicine and indicates the therapeutic potential, with mainly anti-inflammatory, antimicrobial, antinociceptive, and analgesic properties.

The broad-spectrum antimicrobial effects of the *C. albidus* extracts summarized in this review support further exploration of the extracts and their components for novel approaches. Furthermore, its potential to regulate physiological processes in the nervous system makes this species an interesting candidate to be used for neurodegenerative disorders and diseases, especially in those diseases with an inflammatory or oxidative implication.

No references to metabolites that could exhibit toxicity have been found, nor are there known episodes of intoxication in humans. Based on ethnobotanical research, the paraphyletic varieties of the *Cistus* subgenus, especially *C. creticus* and *C. albidus*, were traditionally used for direct consumption of their leaves (dried and ground) or their extracts (as infusions and decoctions). Therefore, based also on its long traditional use, this plant could be considered safe.

In this work, it has been argued that the effectiveness of traditional preparations based on *C. albidus* may be based mainly on three factors. On the one hand, there is the pharmacological activity that derives from certain terpenes and polyphenols naturally present in *C. albidus*. On the other hand, increased bioavailability of phenolic and terpenoid compounds is suggested due to the presence of *α*-bisabolol, tocopherols, and fatty acids. Finally, since they are present in the same drug (aerial parts), all the compounds coexist in an equilibrium, which suggests a natural synergism. Hence, the entire drug should be more effective than the isolated substances that compose it. This natural composition of all the compounds found in this species makes *Cistus albidus* a medicinal plant with high potential, especially for treating and preventing neurodegenerative diseases and disorders.

We hope that this review will help lay the groundwork for further comprehensive pharmacological and pharmaceutical studies to better understand the clinical relevance and use of *C. albidus* and improve its legal status as a traditional medicinal plant.

## Data Availability

Not applicable.
